# Public perceptions and practices on air quality and respiratory health: insights from a cross-sectional study in Saudi Arabia

**DOI:** 10.25122/jml-2024-0385

**Published:** 2025-04

**Authors:** Turki Alanzi, Norah Aljarbooa, Faris AlSalem, Rahaf Sawan, Bushra Albalawi, Gumashah Ababtain, Reema Taha, Mirnan Toonsi, Mohammed Aloufi, Husain Alsharifa, Joud Alshangiti, Nouf Alanzi

**Affiliations:** 1 Imam Abdulrahman Bin Faisal University, Dammam, Saudi Arabia; 2 College of Pharmacy, Qassim University, Al-Qassim, Saudi Arabia; 3 College of Pharmacy, King Khalid University, Abha, Saudi Arabia; 4 College of Pharmacy, Jazan University, Saudi Arabia; 5 Laboratory Department, Maternity and Children Hospital, Tabuk, Saudi Arabia; 6 Mohammed Almana College for Medical Sciences, Eastern Province,Saudi Arabia; 7 College of Pharmacy, Taibah University, Madinah Al Munawwarah, Saudi Arabia; 8 College of Pharmacy, Umm Al-Qura University, Makkah, Saudi Arabia; 9 College of Medicine, King Saud University, Riyadh, Saudi Arabia; 10 College of Medicine and Medical Sciences, Arabian Gulf University, Manama, Bahrain; 11 College of Medicine, Fakeeh College for Medical Science, Jeddah, Saudi Arabia; 12 Jouf University, Al-Jouf, Saudi Arabia

**Keywords:** air quality, respiratory health, public perception, environmental awareness, Saudi Arabia

## Abstract

This study aimed to assess public perceptions and practices regarding air quality and its impact on respiratory health in Saudi Arabia. A cross-sectional survey was conducted among 539 participants, selected through stratified random sampling across urban, semi-urban, and rural areas. Data were collected via an online questionnaire and analyzed using descriptive statistics, *t*-tests, and ANOVA. Findings indicated that 63.2% (*n* = 341) of participants occasionally checked air quality reports, with moderate confidence in interpreting them (52.5%, *n* = 283). Awareness of the health impacts of air pollution was higher in urban areas, while rural participants showed less concern and lower awareness (*P* < .0001). Younger participants (18-30 years) had the highest concern about air pollution (mean: 3.39), whereas older participants demonstrated lower awareness of associated health risks (*P* < .0001). The study found no significant gender differences in perceptions (*P* > .05). Despite moderate concern about air pollution, participants perceived a low impact on their quality of life (mean: 2.85). The study underscores the importance of localized public health strategies to address air quality concerns and mitigate respiratory health risks in Saudi Arabia.

## INTRODUCTION

Air quality has emerged as a pressing global health concern, driven by rapid urbanization, industrial activities, and vehicular emissions [1,2]. The escalating levels of air pollution have serious consequences for human health, particularly respiratory health, which has captured the attention of researchers, policymakers, and public health officials [3-8]. Exposure to pollutants such as particulate matter (PM2.5 and PM10), nitrogen dioxide (NO2), sulfur dioxide (SO2), and ozone (O3) has been strongly linked to respiratory conditions, including asthma, chronic obstructive pulmonary disease (COPD), and bronchitis [9,10]. According to the World Health Organization (WHO), millions of premature deaths annually are attributed to air pollution-related respiratory diseases. For instance, in 2021, air pollution caused 8.1 million deaths globally, making it the second leading cause of death, including among children under five [11].

Saudi Arabia exemplifies the critical nature of this issue. Its air quality is deemed hazardous by WHO standards, with a yearly PM2.5 concentration averaging 88 µg/m^3^, far exceeding the recommended limit of 10 µg/m^3^ [12]. The annual mortality rate attributed to PM2.5 in Saudi Arabia stands at 8,536, accounting for 9% of the total yearly deaths. Furthermore, in 2017, PM2.5 exposure resulted in 315,200 disability-adjusted life years (DALYs), ranking ambient particulate matter as the seventh most significant health risk factor in the country. This air pollution burden is closely tied to chronic diseases such as cancer, asthma, and respiratory illnesses [13-17].

Despite the well-documented health effects of air pollution, there is a notable gap in understanding public perceptions of air quality and associated health behaviors [1,3]. Scientific studies confirm a strong link between air pollution and respiratory health [16], but how the public perceives these risks and translates them into actions is less understood. Public perception is pivotal, as it shapes health behaviors, drives preventive actions, and fosters support for policies to improve air quality. However, perceptions are influenced by individual awareness, media coverage, cultural beliefs, and personal experiences. Understanding how the Saudi public perceives air quality and its health impact is vital for designing effective interventions. Public cooperation is essential for the success of policy measures to reduce air pollution. This research aims to bridge the gap between scientific knowledge and public understanding, ultimately enhancing air quality management efforts.

A crucial yet underexplored area involves public practices related to managing air quality at the individual level. While governmental interventions are critical, individual actions such as using air purifiers, wearing masks, avoiding outdoor activities during high pollution, and advocating for cleaner environments can significantly mitigate respiratory health risks [18]. However, little research systematically explores how individuals perceive air pollution risks and their actions to protect themselves. Awareness of air quality issues varies widely among populations and is influenced by education, access to information, and socio-economic status. Studies indicate that individuals with higher education levels are generally more aware of air pollution and its health impacts [19,20]. Moreover, urban residents exhibit greater awareness, where pollution is often more pronounced than rural counterparts [21]. However, awareness does not always translate into concern or action. For example, one study highlighted that while many individuals are aware of air pollution, they often underestimate its severity and associated health risks [22].

Perception of health impacts plays a critical role in determining behavioral responses. Research shows that individuals who recognize a direct link between air quality and respiratory health are likelier to adopt precautionary measures, such as reducing outdoor activities during high-pollution periods [9,10]. However, among lower socio-economic groups, there often exists a disconnect between awareness and perceived health risks, leading to reduced engagement in protective behaviors [23]. This gap highlights the need for targeted education and communication strategies.

External factors, such as government advisories and media coverage, also shape behavioral responses. A study revealed that individuals perceiving higher personal risk from air pollution are more likely to use air purifiers and masks [24]. However, skepticism about the effectiveness of these measures and cultural norms can hinder the consistent adoption of protective behaviors. This underscores the importance of addressing social and cultural factors alongside awareness campaigns.

Government and community initiatives are pivotal in influencing public responses to air quality challenges. Studies demonstrate that public health campaigns and regulations significantly enhance awareness and encourage protective behaviors [25-29]. For example, the 'Clean Air Act' advocacy in the U.S. and 'Smog Alerts' in European cities have mobilized public action effectively [30,31]. Such collaborative approaches between governments and communities are instrumental in combating air pollution.

Policy advocacy also plays a crucial role in improving air quality and public health outcomes. Advocacy for stricter emission standards and renewable energy adoption has yielded significant benefits [32]. Public support for these policies depends on awareness of pollution's health impacts and trust in the policies' efficacy. Therefore, continuous public education and transparent communication from policymakers are essential to fostering widespread support.

Addressing public perceptions and practice gaps is essential for effective public health interventions. Identifying discrepancies between scientific evidence and public understanding can help tailor health communication strategies to dispel misconceptions and encourage healthier behaviors. Additionally, insights into public practices can inform policymakers about community engagement levels and the need for educational programs promoting proactive measures.

This research aimed to comprehensively understand public perceptions and practices regarding air quality and respiratory health in Saudi Arabia. By addressing the gaps identified, the study seeks to offer insights into designing effective public health campaigns and policies to engage communities, promote healthier behaviors, and reduce respiratory diseases linked to poor air quality. Enhanced public cooperation and targeted interventions can significantly improve air quality and public health outcomes.

## MATERIAL AND METHODS

A cross-sectional survey design was adopted in this study. The selection of different approaches and the procedures adopted are explained in the following sections.

### Study settings and participants

This study was conducted in Saudi Arabia, a country that has experienced rapid urbanization and industrial growth in recent decades. These developments and the region's arid climate have contributed to varying levels of air pollution across different regions, making it an ideal setting for examining public perceptions and behaviors related to air quality and respiratory health. The research was carried out across multiple regions in Saudi Arabia, including major urban centers such as Riyadh, Jeddah, and Dammam, as well as selected rural areas. These locations were chosen to capture diverse environments with varying air quality levels, from heavily industrialized urban areas to less polluted rural settings. The study aimed to comprehensively understand how different environmental contexts influence public awareness, perceptions, and behaviors concerning air quality and respiratory health.

The study targeted a diverse cross-section of the Saudi population to ensure that findings are representative of the broader public. The participants were adults aged 18 years and above who were residing in the country.

### Inclusion criteria

The inclusion criteria for this study required participants to be 18 years or older to ensure they could provide informed consent and had the cognitive maturity to understand and respond to the survey questions. Additionally, participants had to be residents of the selected urban or rural areas in Saudi Arabia. This criterion ensured that respondents were familiar with the air quality conditions and environmental issues in their localities. Lastly, language proficiency in Arabic or English was required, as the survey instruments were administered in these languages. This criterion ensured that participants fully comprehended the questions and provided accurate responses.

### Exclusion criteria

Individuals under the age of 18 were excluded from the study to comply with ethical guidelines related to informed consent and to focus on adults who are likely to make independent health-related decisions. Also, non-residents of the selected study areas were excluded to maintain the relevance of the data to the specific environmental contexts of Saudi Arabia.

### Sampling and recruitment

A stratified random sampling method [33] was employed in this study to ensure a representative sample of the Saudi Arabian population across different regions and demographic groups. The sample was stratified based on key variables, including geographic location (urban vs semi-urban vs. rural), age, gender, and education. This approach ensured that all significant subgroups within the population were adequately represented in the study. Within each stratum, participants were randomly selected to reduce selection bias and increase the generalizability of the findings.

Participants were recruited via an online survey distributed through email networks, social media platforms, and community forums. These channels were chosen for their broad reach and ability to engage individuals from various geographic and socioeconomic backgrounds. For large populations, the sample size was calculated using Cochran's formula [34], which is widely used in cross-sectional studies to ensure statistical validity and reliability.

### Sample size calculation

Cochran’s formula is given by:

*n*0 = (Z^2^*p*(1 – *p*))/*e*^2^

where:

- n0: Initial sample size
- Z: Z-value for the desired confidence level (1.96 for 95%)
- p: Estimated proportion of the population with the attribute of interest (assumed at 50% to maximize variability)
- e: Margin of error (5%, or 0.05)

Using this formula, the calculated sample size was 384. Given that the target population—adult Saudi Arabian residents—is very large, a finite population correction was not applied. To account for non-responses and incomplete surveys, the sample size was increased by 40%, resulting in a target of approximately 500 participants. This adjusted sample size was sufficient to detect significant differences across geographic areas and demographic groups.

### Questionnaire design

The initial questionnaire was drafted based on a thorough review of existing studies on air quality perceptions, health impacts, and environmental behaviors. Relevant themes were identified and incorporated, including demographic information, air quality awareness, perceived health impacts, behavioral responses, and views on government and community actions. The draft questionnaire was reviewed by a panel of experts, including two public health specialists and two environmental health researchers from the eHealth department at Imam Abdulrahman bin Faisal University. The experts assessed the content for relevance, clarity, and comprehensiveness, resulting in item wording and structure refinements. To ensure the questionnaire was accessible to participants in Saudi Arabia, it was translated from English to Arabic by a certified professional translator [35]. The Arabic version was then reviewed by bilingual experts to ensure linguistic and cultural appropriateness. Back-translation was performed to confirm the accuracy of the translation. Minor adjustments were made to improve clarity and contextual relevance. The questionnaire was pilot-tested with a convenience sample of 12 participants representing diverse demographic and geographic backgrounds. The pilot study assessed the clarity, reliability, and internal consistency of the questionnaire items. Feedback from participants led to minor modifications, such as rephrasing ambiguous questions and adjusting response options for improved understanding. The Cronbach's alpha coefficient was calculated for each questionnaire section during the pilot study to assess internal consistency. All sections achieved a Cronbach’s alpha score exceeding 0.7, indicating robust reliability [36]. Additionally, the questionnaire’s content validity was ensured by aligning items with the study’s objectives and expert recommendations.

The final questionnaire consisted of six sections: demographic information, air quality awareness, perceived impact on respiratory health, behavioral responses, perception of government and community actions, and concluding insights, which are explained below:

**Demographic information** (Section 1): gathered basic participant information, including age, gender, education level, occupation, and residential area. This data helped segment the respondents to analyze how demographic factors influence perceptions and behaviors related to air quality.

**Air quality awareness** (Section 2): focused on understanding the level of concern participants had about air pollution, how frequently they monitored air quality reports, and their ability to interpret these reports. This section identified the extent of awareness and the factors that shaped participants' understanding of air quality issues.

**Perceived impact on respiratory health** (Section 3): explored participants’ beliefs about the health effects of air pollution, particularly on vulnerable groups. It also assessed their awareness of local initiatives addressing air quality and their perception of how pollution affects their quality of life (QoL). This section linked awareness to perceived health risks and community well-being.

**Behavioral responses** (Section 4): examined the specific actions participants took in response to air pollution, such as using air purifiers or advocating for policies. It also investigated participants' willingness to relocate due to air quality concerns and their level of information about the health risks associated with poor air quality.

**Perception of government and community actions** (Section 5): assessed participants' views on the effectiveness of government efforts to control air pollution and the accountability of local businesses or industries. It also explored the role of individuals and communities in improving air quality, highlighting the perceived responsibility and effectiveness of various stakeholders.

**Final thoughts** (Section 6): served as a concluding section, determining participants’ overall concern about the long-term effects of air pollution on health, their interest in engaging with community programs, and their level of information on reducing exposure to air pollution. This section also invited open-ended responses for suggestions, offering deeper insights into public sentiment and recommendations.

### Data collection

Data for this study were collected over 4 weeks using a structured survey questionnaire in English and Arabic. The questionnaire was distributed exclusively online to ensure broad accessibility and convenience for participants across various regions in Saudi Arabia, including urban, suburban, and rural areas. The survey link was disseminated via popular social media platforms (e.g., WhatsApp, Twitter, and Facebook), academic mailing lists, and community forums. To maximize reach and participation, the research team collaborated with local organizations, community leaders, and online influencers to share the survey link with diverse groups. The survey was hosted on a secure online platform (SurveyMonkey), ensuring easy access and data security.

To ensure data integrity and prevent duplicate responses, the survey platform allowed only one submission per IP address, minimizing the likelihood of multiple entries from the same device. Participants were also required to provide an email address, which was cross-checked to identify and exclude duplicate entries. Additionally, during the analysis phase, the dataset underwent thorough cleaning, where responses were screened for consistency and completeness. Duplicate submissions and incomplete surveys were removed, resulting in a final sample of 539 participants from the initial 586 responses collected.

### Data analysis

Data was analyzed using the Statistical Package for the Social Sciences (SPSS, IBM Version 24). Descriptive statistics, including means and standard deviations, were used to present the demographic characteristics of the participants. Additionally, a two-sample *t*-test with unequal variances and a one-way ANOVA were conducted to analyze the data further.

### Control for confounding factors

Potential confounding factors were considered during both the study design and analysis phases to ensure the validity of the study's findings. Key demographic variables, including age, gender, education level, and geographic location (urban, semi-urban, rural), were identified as potential influencers of participants' awareness, perceptions, and behaviors related to air quality and respiratory health. A stratified random sampling method was employed to address these variables, ensuring proportional representation across demographic groups and geographic regions to minimize sampling bias. The survey instrument was designed to collect detailed demographic data, allowing for identifying and analyzing these factors in relation to the study outcomes. During data analysis, statistical methods such as *t*-tests and ANOVA were used to examine differences across demographic groups while controlling for the influence of other variables. This approach allowed the study to account for confounding factors and provide a clearer understanding of the relationships being investigated.

## RESULTS

Participants were predominantly young adults, with 47.68% aged 18-30 years and a smaller proportion in older age groups (Table 1). In terms of educational level, the majority had at least a diploma (32.10%) or a bachelor's degree (37.48%), while fewer participants had a master's degree (8.35%) or reported having no formal education (6.49%). The gender distribution was skewed towards men (59.55%), with women comprising 40.45% of the sample. In terms of residence, the majority lived in urban areas (42.49%), followed by semi-urban (27.27%) and rural areas (30.24%).

**Table 1:** Participant demographics

Variables		*n*	Relative frequency
Age	18-30	257	47.68%
	31-40	122	22.63%
	41-50	91	16.88%
	>50	69	12.80%
Education	No education	35	6.49%
	Primary/Secondary level	84	15.58%
	Diploma	173	32.10%
	Bachelor’s degree	202	37.48%
	Master’s degree	45	8.35%
Gender	Female	218	40.45%
	Male	321	59.55%
Residence	Rural	163	30.24%
	Semi-urban	147	27.27%
	Urban	229	42.49%

Most participants (63.2%) reported checking air quality reports occasionally, while 21.5% did so weekly, 6.8% daily, 3.7% monthly, and 4.8% never. The Air Quality Index (AQI) was used by 53.6% of participants to assess air quality, whereas 82.7% relied on visual indicators such as smog and haze, and 23.6% used local news reports. Despite this, 52.5% expressed only moderate confidence, and 27.8% admitted to being not very confident in interpreting air quality data, highlighting a lack of awareness in accurately assessing air pollution levels. Most participants believed that the elderly (41.7%) and people with respiratory disorders (36.5%) were the most vulnerable to the health effects of air pollution. However, fewer recognized pregnant women (5.6%) and children (4.9%) as at-risk groups. Most responders (73.6%) preferred closing windows or using indoor air filters, followed by air purifiers or masks (32.4%), avoiding outside activities (17.3%), and public transportation or carpooling (12.9%). Despite concerns about air quality, 83.5% of participants stated that they had not considered relocating due to pollution. Furthermore, 62.8% believed that the health effects of air pollution, particularly on respiratory health, were not widely understood. 58.9% of participants supported strict industry air pollution regulations even if it would raise consumer prices.

Nearly two-thirds (65.3%) of participants said government air pollution management initiatives were moderately effective, 21.6% rated them as highly effective, and 12.3% considered them slightly effective. Most participants (51.8%) felt that local firms or industries should be liable for air pollution. Most participants occasionally discussed air quality and health with friends, family, and coworkers (87.2%). Most participants (78.9%) expressed significant interest in community air quality programs. As summarized in Table 2, participants displayed moderate concern about air pollution, with a mean score of 2.99 out of 5. However, their concern about long-term respiratory health effects was notably higher, with a mean score of 3.89, suggesting that air pollution is perceived as a serious long-term health risk rather than an immediate concern. Participants demonstrated moderate awareness of air pollution-related health risks (mean score: 3.04), indicating a basic level of understanding but a need for further education to enhance public knowledge. The perceived impact of air pollution on overall quality of life was relatively low (mean score: 2.85). Support for air quality improvement policies was moderate, with a mean score of 3.12, indicating a balanced tendency.

**Table 2:** Participants' perceptions of air quality and its effects

Measure	Scale	Mean score
Level of concern about air pollution	1: Not concerned at all; 5: Extremely concerned	2.99
Long-term effects of air pollution on respiratory health	1: Not detrimental at all; 5: Very detrimental	3.89
Awareness of health risks associated with air quality	1: Highly aware; 5: Not at all aware	3.04
Impact on QoL	1: No effect; 5: Greatly effects	2.85
Likeliness of advocating policies aimed at improving air quality	1: Very unlikely; 5: Very likely	3.12

The *t*-test results (Table 3) indicate no statistically significant differences between male and female participants regarding air quality perceptions and its effects. Both genders had similar levels of concern regarding air pollution, with mean scores of 3.06 for women and 2.95 for men (*P* = .2869). Similarly, their perspectives on the long-term respiratory health consequences of air pollution (women: 3.83, men: 3.93, *P* = .2138) and health risk awareness (women: 3.07, men: 3.02, *P* = .6401) were comparable. Although women reported a slightly greater perceived impact of air pollution on quality of life (mean = 2.76) than males, the *P* value of 0.0604 indicates that this difference was not statistically significant. Finally, gender did not significantly affect air quality policy advocacy (women: 3.09, men: 3.14, *P* = .6884).

**Table 3:** Gender-based differences in air quality perceptions and policy support

Factors	Gender	*n*	Mean	Variance	*P*
Level of concern about air pollution	Female	218	3.06	1.42	.2869
	Male	321	2.95	1.53	
Long-term effects of air pollution on respiratory health	Female	218	3.83	0.89	.2138
	Male	321	3.93	0.80	
Awareness of health risks associated with air quality	Female	218	3.07	1.55	.6401
	Male	321	3.02	1.45	
Impact on QoL	Female	218	2.97	1.61	.0604
	Male	321	2.76	1.60	
Likeliness of advocating policies aimed at improving air quality	Female	218	3.09	1.54	.6884
	Male	321	3.14	1.74	

The ANOVA results (Table 4) showed significant differences in participants' opinions of air pollution based on residential location. Rural residents had lower concern for air pollution (mean: 2.76) than semi-urban (3.17) and urban (3.15) residents (*P* = .0082). Similarly, awareness of air quality-related health risks was lowest among rural participants (mean: 2.50) compared to semi-urban (mean: 3.25) and urban (mean: 3.28), with a significant *P* value of <.0001. The impact of air pollution on QoL was significantly lower in rural areas (mean: 2.25) compared to semi-urban (mean: 3.22) and urban (mean: 3.04), with a *P* value <.0001. The perceptions regarding the long-term consequences of air pollution on respiratory health were not significantly different (*P* =.4671). Semi-urban residents were slightly more likely to advocate for air quality improvement policies than rural and urban residents (*P* = .0484).

**Table 4:** Differences between participants' perceptions based on their place of living

Factors	Residence	*n*	Mean	Variance	*P* value
Level of concern about air pollution	Rural	163	2.76	1.43	.0082*
	Semi-urban	147	3.17	1.43	
	Urban	229	3.05	1.51	
Long-term effects of air pollution on respiratory health	Rural	163	3.95	0.71	.4671
	Semi-urban	147	3.82	0.80	
	Urban	229	3.90	0.94	
Awareness of health risks associated with air quality	Rural	163	2.50	0.94	< .0001*
	Semi-urban	147	3.25	1.44	
	Urban	229	3.28	1.63	
Impact on QoL	Rural	163	2.25	1.15	< .0001*
	Semi-urban	147	3.22	1.38	
	Urban	229	3.04	1.72	
Likeliness of advocating policies aimed at improving air quality	Rural	163	3.17	1.64	.0484
	Semi-urban	147	3.29	1.50	
	Urban	229	2.97	1.74	

* Statistically significant difference at .05 confidence interval

The ANOVA results (Table 5) reveal significant age-related variations in air quality perceptions across all measured components. Younger participants (18-30 years) had the highest level of concern about air pollution (mean: 3.39), with concern declining significantly among older age groups, particularly 41–50 years (mean: 2.14) and those over 50 (mean: 2.42) (*P* < 0.0001). Perceived long-term respiratory health consequences were also higher among younger participants (mean: 3.99) than older groups, although the differences were less evident (*P* = .0159). Similarly, awareness of air pollution-related health risks was highest among younger individuals (mean: 3.30), dropping considerably with age, especially among those aged 41-50 (mean: 2.67) and over 50 (mean: 2.71), with a *P* <.0001. The perceived impact of air pollution on QoL followed the same trend, with younger individuals reporting the highest impact (mean: 3.12), while participants aged 41–50 (mean: 2.13) and over 50 (mean: 2.42) rated the impact significantly lower (*P* < 0.0001). Regarding policy advocacy, younger participants were more likely to advocate for air quality policies (mean: 3.63) than older groups (41-50, over 50) (*P* <.0001).

**Table 5:** ANOVA results assessing the difference between participants' perceptions based on their age

Factors	Age	*n*	Mean	Variance	*P*
Level of concern about air pollution	18-30	257	3.39	1.46	< .0001*
	31-40	122	3.13	1.27	
	41-50	91	2.14	0.66	
	>50	69	2.42	1.22	
Long-term effects of air pollution on respiratory health	18-30	257	3.99	0.87	.0159*
	31-40	122	3.87	0.59	
	41-50	91	3.64	0.97	
	>50	69	3.91	0.85	
Awareness of health risks associated with air quality	18-30	257	3.30	1.37	< .0001*
	31-40	122	2.95	1.60	
	41-50	91	2.67	1.36	
	>50	69	2.71	1.39	
Impact on QoL	18-30	257	3.12	1.86	< .0001*
	31-40	122	3.05	1.34	
	41-50	91	2.13	0.76	
	>50	69	2.42	1.16	
Likeliness of advocating policies aimed at improving air quality	18-30	257	3.63	1.55	< .0001*
	31-40	122	3.14	1.28	
	41-50	91	2.24	0.90	
	>50	69	2.33	1.14	

* Statistically significant difference at .05 confidence interval

The ANOVA results in Table 6 indicate that education level does not significantly influence air quality perceptions across any of the examined categories. Concern about air pollution was slightly higher among participants with no formal education (mean: 3.23) and those with a master’s degree (mean: 3.07), but the difference was not statistically significant (*P* = 0.2704). Perceptions of the long-term impacts of air pollution on respiratory health were similar across education levels, with mean scores ranging from 3.79 to 4.03 (*P* = .6458). Participants with a master’s degree had slightly lower awareness of air quality-related health risks (mean: 2.84); however, this variation was not statistically significant (*P* = 0.8513). The impact on QoL and the likelihood of lobbying for air quality improvement legislation did not differ by education level, with *P* values of.4517 and.6014, respectively.

**Table 6:** ANOVA results assessing the difference between participants' perceptions based on their level of education

Factors	Education	*n*	Mean	Variance	*P*
Level of concern about air pollution	No education	35	3.23	1.65	.2704
	Primary/Secondary level	84	2.89	1.30	
	Diploma	173	3.11	1.58	
	Bachelor’s degree	202	2.89	1.53	
	Master’s degree	45	3.07	1.11	
Long-term effects of air pollution on respiratory health	No education	35	4.03	0.73	.6458
	Primary/Secondary level	84	3.79	0.77	
	Diploma	173	3.94	0.85	
	Bachelor’s degree	202	3.88	0.95	
	Master’s degree	45	3.87	0.48	
Awareness of health risks associated with air quality	No education	35	3.11	1.99	.8513
	Primary/Secondary level	84	3.04	1.53	
	Diploma	173	3.06	1.51	
	Bachelor’s degree	202	3.05	1.35	
	Master’s degree	45	2.84	1.63	
Impact on QoL	No education	35	3.03	1.50	.4517
	Primary/Secondary level	84	2.88	1.31	
	Diploma	173	2.90	1.76	
	Bachelor’s degree	202	2.72	1.67	
	Master’s degree	45	3.00	1.41	
Likeliness of advocating policies aimed at improving air quality	No education	35	2.91	2.08	.6014
	Primary/Secondary level	84	3.08	1.47	
	Diploma	173	3.21	1.67	
	Bachelor’s degree	202	3.06	1.74	
	Master’s degree	45	3.24	1.28	

## DISCUSSION

This study sheds light on Saudi air quality and respiratory health attitudes and practices. The ANOVA and *t*-test results help explain how demographics affect these beliefs and behaviors. The *t*-test showed no significant variations in air quality and effect perceptions between men and women. This supports earlier research indicating that gender does not greatly alter environmental perceptions, especially in air pollution-affected areas [22]. This study found that men and women were equally concerned about air pollution and respiratory health. Due to significant air pollution in Saudi Arabia, air quality is a concern for all genders. The lack of significant gender disparities means public health initiatives can focus on community-wide techniques without gender-specific tailoring.

However, ANOVA results showed substantial disparities by participant residency. Rural residents were less concerned about air pollution and its health consequences than urban and semi-urban ones [37]. This gap is consistent with the research that shows urban inhabitants, who are more exposed to visible pollution sources, are more concerned about air quality [21]. Rural inhabitants may be less concerned about air pollution due to poor visibility or a lack of information about its health effects [38]. This suggests that rural areas need targeted air quality education and outreach activities to improve awareness of health risks.

Our results showed that different age groups had distinctive views. Air pollution concern was higher among younger people, notably 18–30-year-olds. This concern did not always convert into preventative measures like avoiding outdoor activities during high pollution or utilizing air purifiers. This tendency supports earlier research showing that younger people are more aware of environmental issues but less likely to take health precautions [19,37-39]. The perceived invulnerability of younger populations and the lack of immediate health repercussions that might modify behavior may explain this gap. Our analysis showed significant differences in air quality health risk knowledge across age groups. Younger people were more aware than older participants, especially those over 50. Since older persons are more likely to have respiratory illnesses aggravated by poor air quality, this discovery is worrying. Targeted educational initiatives to reach vulnerable groups like elderly populations may be needed due to decreased access to information or health communication campaigns.

All subjects reported low perceived QoL impacts from air pollution despite high concern. This shows a gap between air pollution understanding and its perceived consequences on daily living. Other research has found that people acknowledge air pollution but do not completely understand its effects [23]. This could be due to a lack of obvious health symptoms, the normalization of bad air quality, or cultural values that emphasize other considerations over environmental health. This perceived gap may require targeted communication techniques that directly link air quality to health outcomes and quality of life.

The study also indicated that many participants supported strict industry air pollution laws, even if they increased consumer costs. This finding supports global trends toward environmental regulation and a growing public awareness of the need for systemic measures to reduce air pollution [32]. The willingness to pay more for better air quality shows a solid policy advocacy foundation in Saudi Arabia. Public health campaigns emphasizing the long-term health and economic benefits of cleaner air could use this support to push for stricter environmental legislation.

The large residence-based perception variations emphasize the need for specialized public health initiatives. Urban and semi-urban citizens, who showed more care and awareness, may be more responsive to community-based activities and policy lobbying, while rural communities may need more education. This stratification of public attitudes emphasizes the need for community-specific treatments.

This work adds to the theoretical knowledge of environmental health behavior, specifically demographic influences on air quality perceptions and actions. The considerable age-related and residential differences in awareness and concern emphasize the importance of demographic variables in health behavior and environmental risk perception models. These findings suggest that public health interventions should target awareness and behavior gaps in older persons and rural residents. Policymakers can improve the respiratory health of the population by creating focused information programs and offering accessible resources.

One of the key strengths of this study is its robust sampling method, using stratified random sampling to ensure the inclusion of diverse demographic groups from urban, semi-urban, and rural areas across Saudi Arabia. This approach provides a comprehensive representation of the population and enhances the generalizability of the findings. Additionally, the study utilized an online survey platform, which ensured broad accessibility and convenience for participants across various regions. The questionnaire was well-designed, carefully translated, and pilot-tested to ensure reliability and cultural relevance. Furthermore, rigorous statistical analyses, including *t*-tests and ANOVA, allowed for a thorough examination of the data, helping to uncover meaningful insights into public perceptions and behaviors related to air quality and respiratory health.

However, shortcomings should be noted in this work. First, self-reported data may be biased by social desirability or recollection bias, impacting reported behaviors and views. Second, the cross-sectional study design makes it difficult to link demographic characteristics to environmental health behaviors. The sample may not be representative of the population, especially geographically, limiting its generalizability. To confirm and expand these findings, long term and diverse studies are needed.

## CONCLUSION

In conclusion, this study provides valuable insights into the public's perceptions and practices regarding air quality and respiratory health in Saudi Arabia. The findings suggest that while there was a moderate level of concern about air pollution, there is a need for more targeted education, particularly in rural areas, and communication strategies that better connect air quality to daily life and health outcomes. The support for stricter industrial regulations presents an opportunity for policymakers to advance more aggressive environmental policies. Future research should continue to explore these perceptions and behaviors, particularly in light of ongoing urbanization and industrialization in Saudi Arabia, to inform more effective public health interventions.
